# Human and mouse brain-derived endothelial cells require high levels of growth factors medium for their isolation, *in vitro* maintenance and survival

**DOI:** 10.1186/2045-824X-5-10

**Published:** 2013-05-14

**Authors:** Stefania Elena Navone, Giovanni Marfia, Sara Nava, Gloria Invernici, Silvia Cristini, Sergio Balbi, Simone Sangiorgi, Emilio Ciusani, Alessandra Bosutti, Giulio Alessandri, Mark Slevin, Eugenio Agostino Parati

**Affiliations:** 1 Laboratory of Cellular Neurobiology, Cerebrovascular Diseases Unit, IRCCS Foundation Neurological Institute “C. Besta”, via Celoria 11, Milan, 20133, Italy; 2 Unità Produttiva per Terapie Cellulari (UPTC), IRCCS Foundation Neurological Institute “C. Besta”, Milan, Italy; 3 Department of Surgical Sciences, Neurosurgical Unit, University of Insubria, Varese, Italy; 4 Laboratory of Clinical Investigations, IRCCS Foundation Neurological Institute “C. Besta”, Milan, Italy; 5 School of Healthcare Science, Manchester Metropolitan University, Manchester, United Kingdom

**Keywords:** Brain microvascular endothelial cells, Endothelial permeability, Endothelial junctions, CD31, Blood brain barrier

## Abstract

**Background:**

Brain microvascular endothelial cells (BMVECs) constitute the primary limitation for passage of ions and molecules from the blood into the brain through the blood brain barrier. Numerous multi-step procedures for isolating and culturing BMVECs have been described. However, each one demonstrates major limitations in purity of culture and/or low proliferation rate. Our goal was to study the efficiency of our pending patent medium, Endothelial Proliferation Medium (EndoPM), on the isolation and purification of human and murine BMVECs.

**Methods:**

BMVECs, cultured in EndoPM were compared to those cultured in a commercial medium EBM. Cultures were characterized by flow cytometric analysis, lineage differentiation, the ability to form tube-like structure, immunofluorescence, molecular analyses and also in an *in vivo* model assay. Moreover permeability was assayed by monitoring the passage of Dextran-FITC through a tight monolayer of BMVECs grown to confluence in Boyden chambers. One way Anova two-tailed test was utilized for all statistical analyses.

**Results:**

The properties of ECs in human and murine BMVECs is confirmed by the expression of endothelial markers (CD31, CD105, CD146, Tie-2 and vWF), of representative proangiogenic genes (ICAM1, VCAM1 and integrin ITGAV), of considerable tube-forming ability, with low-density lipoprotein uptake, eNOS and GLUT-1 expression. Furthermore cells are able to express markers of the junctional architecture as VE-cadherin, β-catenin and Claudin-5 and greatly reduce dextran permeability as barrier functional test. Moreover BMVECs spontaneously organize in vascular-like structures and maintain the expression of endothelial markers in an *in vivo* xenograft model assay. The significant effect of EndoPM is confirmed by the study of proliferation index, survival index and the behaviour of BMVECs and fibroblasts in co-culture conditions.

**Conclusion:**

Herein we describe a simple and reproducible method for the isolation and expansion of human and mouse BMVECs, based on a newly formulated medium (EndoPM) with optimized concentration of growth factors (EGF, FGF-2 and Bovine Brain Extract-BBE). This procedure should facilitate the isolation and expansion of human and mouse BMVECs with extended lifetime, good viability and purity. This approach may provide an effective strategy to aid phenotypical and functional studies of brain vessels under physiological and pathological conditions.

## Background

Angiogenesis is a hallmark of diverse pathologies, such as cancer, atherosclerosis and cerebrovascular diseases [1,2]. The progression of all these diseases depends also on the formation of new blood vessels; hence, there is substantial interest in the specific features of endothelial cells (ECs) during the development of these pathologies. The growing evidence that abnormal ECs frequently accompany organ dysfunction and disease [3,4], has led to a strong interest in the development of model systems both *in vivo* and *in vitro* to study EC characteristics. ECs comprise only 1–2% of the total number of cells in tissues [2], therefore methods for their purification and subsequent characterization are highly desirable.

Brain microvascular endothelial cells (BMVECs) form the major element of the blood-brain barrier (BBB) and constitute the primary limitation for passage of substances, both soluble and cellular, from the blood into the brain [5]. They possess unique morphological and functional characteristics that distinguish them from organs- and tissue-endothelium [6]. To perform their physiological function, BMVECs utilize unique features such as specialized tight junctions and various transporters for vitamins, nutrients and metabolic precursors, helping to maintain brain homeostasis [7].

Various multi-step procedures for isolating and culturing BMVECs derived from different species [8] are available, with both advantages and limitations [9,10].

While primary BMVECs cultures retain the closest similarity to phenotypic characteristics of brain endothelium [11], they are extremely time consuming and expensive to generate, and are easily contaminated by other cells. They are difficult to obtain in large numbers, with a good proliferation and viability and free from fibroblasts contamination [12-14].

While immortalized brain EC lines offer a number of investigative advantages, including less time required for their isolation and *in vitro* growth, immortalization involves the introduction of foreign, immortalizing genes (i.e. telomerase and SV40) that may affect a great variety of cellular functions as they may interact with numerous proteins and alter the physiological cell cycle as well as the expression of several proteins and receptors [15].

Here, we describe a new procedure to isolate and cultivate human and murine BMVECs utilizing our pending patent medium, the Endothelial Proliferation Medium called EndoPM (patent number MI2011A 000201) that allows for human and mouse BMVECs isolation and a longer proliferation, particularly when compared with BMVECs cultured in standard commercial available Endothelial Basal Medium (EBM) with growth factors supplement kit. EndoPM is a blend of essential amino acids, inorganic salts and other components, along with an optimized mix of human growth factors where EGF and FGF-2 are at high concentration levels. We demonstrate that BMVECs cultured in EndoPM retain their morphological and functional key characteristics of *in vivo* ECs [16]. Furthermore cells cultured in EndoPM medium are able to express functional markers of tight junction as Claudin-5, as well as adherent junction proteins such as β-catenin and VE-cadherin and greatly reduce dextran permeability as barrier functional test.

In conclusion, our optimized medium supports the culture of established human and mouse BMVECs and improves the efficacy of their isolation and expansion *in vitro* allowing the study of physiological and patho-physiological endothelium.

## Methods

### Isolation of BMVECs

The experimental protocol was approved by the ethics committee of the IRCCS Foundation Neurological Institute “C. Besta” (Milan, Italy) and IRCCS Foundation Ospedale Maggiore Policlinico, Mangiagalli and Regina Elena (Milan, Italy). Human fetal brains were obtained from five 10-12 week-old healthy foetuses, according to the ethical guidelines of the European Network for Transplantation (NECTAR). Tissue specimens were stored in medium DMEM/F12 (Gibco, Grand Island, NY) containing, penicillin 100 U/ml at 4°C for less than 48 h prior to processing.

Procedures involving animals and their care were conducted in conformity with all procedures following institutional guidelines which, in turn, are in compliance with national (D.L. No. 116, G.U. Suppl. 40, Feb. 18, 1992, Circolare No. 8, G.U., 14 Luglio 1994) and international laws and policies (EEC Council Directive 86/609, OJ L 358, 1 Dec.12, 1987; NIH Guide for the Care and use of Laboratory Animals, U.S. National Research Council, 1996). Brains from male CD1 mice (Charles River, Calco, LC, Italy) were removed and stored in DMEM/F12 plus antibiotics until used. After several washes with PBS/antibiotics, tissues were finely minced using surgical scissors and then incubated with Liberase Blendzyme 2 (Roche, Mannheim, Germany) at a concentration of 0.625 Wu/ml at 37°C on a rotator for 2 hours. After enzymatic digestion, the cell suspension was washed with D-PBS, centrifuged at 1200 rpm/10 min and then plated in 25 cm^2^-flask (one for each brain) coated with collagen type I (BD Bioscience, San Diego, CA, USA), in EndoPM, specifically developed by our laboratory (pending patent medium MI2011A 000201) to select and expand ECs. This medium contains very high levels of growth factors (Bovine Brain Extract (6 μg/mL), Fibroblast Growth Factor (5 ng/mL), and Epidermal Growth Factor (10 ng/mL) in particular) and a blend of hormones called Hormone Mix, composed by by apotransferrin (48.82 μg/mL), selenium (2.37 ng/mL), progesterone (2.88 ng/mL), putrescine (48.25 μg/mL), insulin (11.5 μg/mL). Cells were maintained at 37°C, 5% CO_2_. After 24 h of culture, non adherent cells were removed from the flasks and reseeded in a new collagen type I -coated culture 25 cm^2^-flask in 6 mL of fresh EndoPM medium.

The medium was changed every 10 days, the cultured cells were passaged at a split ratio of 1:4 every 14 days and detached by TrypLE Select (Gibco). BMVECs at passages P10-P15 were used as indicated for all experiments. Experiments were performed in triplicates.

Human and Mouse Microvascular Endothelial Cells (HMVECs), cultured in complete EBM medium with growth factors supplement kit (Lonza Group Ltd, Basel Switzerland), were utilized as control samples.

### Survival assay

All experiments were done on 12-well plates, in triplicate for each treatment. Cells were dissociated with TrypLE (Gibco) and diluted to 5 × 10^5^ cells/mL. 5 × 10^5^ cells were added into each well and placed into 5% CO_2_, 37°C incubator. EndoPM or EBM were added to the specified well. At different time points, cells were dissociated with TrypLE and counted by cytometry. For proliferation experiments, media were changed weekly until the day of analysis.

Proliferation index represents the cell number at a specific time point divided by the number of input cells at time 0; survival index represents the number of harvested live cells 12 hours after plating in EndoPM or EBM (Table 1).

**Table 1:** Proliferation index and survival index of human and mouse BMVECs cultured in EndoPM or EBM

	**Human**		**Mouse**	
	**EndoPM**	**EBM**	**EndoPM**	**EBM**
**proliferation index**	1087,77 ± 105	422,96 ± 40,13	666 ± 53,2	372,22 ± 35,3
**survival index**	102,05 ± 3,04	36,85 ± 12,03	98,53 ± 4,13	49,15 ± 10,2

### Nutrient starvation

Human and mouse BMVECs were incubated in growth media at 37°C 5% CO_2_ in a humidified atmosphere, in a 6-wells culture plate, 3 × 10^5^ cells per well. When 80% confluence was reached, cells were washed twice in PBS and incubated overnight in starvation medium composed of DMEM/F12 (Gibco), 1% L-Glutamine (Gibco), 0.5% FBS, to induce cells synchronization.

The day after, starved cells were treated with complete EndoPM or EBM medium for 5h or overnight. Cells were than detached with TripLE (Gibco) following manufacturer instruction and processed for cell cycle analysis.

### Cell cycle analysis

Cell cycle was evaluated by flow cytomety analysis after synchronization in starvation medium. After the synchronization time, starved cells were treated with complete EndoPM or EBM medium for 5 h or overnight. 5 × 10^5^ human or mouse BMVECs cells were fixed in 1 ml cold ethanol (70% vol/vol in PBS) with gentle vortexing (about 5 s) to obtain a mono-dispersed cell suspension, and maintained in ethanol, at 4°C, for at least 2 h. After ethanol fixation, cells were centrifuge 1200 rpm/10 min and the ethanol thoroughly decanted. The pellet was then re-suspended in 500 μL of PBS and incubated with 1 mg/mL RNAse A, at 37°C for 15 minutes. Cells were centrifuge for 1200 rpm /10 min and the supernatant discarded. The pellet was then resuspended in 500 μL of PBS with the addition of 50 μg/mL of Propidium iodide (PI) staining solution. Cells were kept in the dark at 37°C for 20 min and then analysed using a FACScalibur flow cytometer and Cell Quest software (BD Bioscience).

### Co-culture of human BMVECs and fibroblasts (NHDF)

Human BMVECs were cultured in EndoPM or EBM in standard conditions. Normal human dermal fibroblasts (NHDF, Promocell GmbH, Heidelberg, Germany) were cultured in MEM Eagle Medium (Lonza) supplemented with 10% FBS and 1% L-Glutamine. Co-cultures (n = 3) were produced by pooling BMVECS and NHDF with a defined ratio (BMVECS:NHDF = 3:1) in the following medium mix (EndoPM: EMEM medium = 4:1). Fibroblasts were previously labeled with CFDA-SE (Invitrogen, Carlsbad, CA, USA) to permit their identification in co-culture systems. The data were acquired by immunofluorescence.

*Fibroblast quantification*: The quantification of CFDA-SE positive cells was performed by fluorescence microscopy at T0, T7 and T14 (days in culture). Briefly, cells counts were performed on a minimum of 9 independent fields (3 fields/3 coverslips/treatment) of photomicrographs captured with the 40× objective. Total counts of CFDA-SE positive cells were performed and the number of positive cells per culture was expressed as the percentage of the total cells. DAPI supplied the total number of cells. Images were acquired by a Nikon Eclipse TE300 inverted microscope equipped with a Zeiss Axiovision device camera.

### Immunofluorescence and flow cytometry

Human and murine BMVECs were plated on collagen-coated permanox chamber slides (Nunc, Naperville, IL, USA). Cells were fixed and analysed for the presence of endothelial markers by means of immunostainings as previously described [17]. VE-Cadherin, Claudin-5 and β-catenin were detected, according to manufacturer instructions, 5 days after incubations of cells in EndoPM medium without Hormone Mix.

Three separate immunofluorescence analyses were performed on human and murine BMVECs; positive cells were counted in a blind manner.

Human and murine BMVECs were characterized for endothelial marker expression also by means of FACS. The controls were isotype-matched mouse IgG. The cytometric analyses were done with a FACScalibur flow cytometer and Cell Quest software (BD Bioscience). The antibodies used for immunofluorescence and flow cytometry are described in Table 2.

**Table 2:** Human and mouse antibodies utilized for immunofluorescence and flow cytometry analyses

**Antibody**	**Dilution**	**Manufacturer**
**Immunofluorescence**		
**Human**		
CD31	1:50	Sigma
vWF	1:50	Sigma
GLUT-1	1:100	Millipore
e-NOS	1:100	Millipore
UEA-1	1:20	Sigma
hNAg	1:20	Chemicon
Draq 5	1:800	Alexis
VE-cadherin	1:250	Santa Cruz
Claudin-5	1:1000	Invitrogen
β-catenin	1:250	Abcam
**Mouse**		
CD31	1:100	Millipore
vWF	1:50	Santa Cruz Biotechnology
GLUT-1	1:100	Millipore
e-NOS	1:100	Millipore
**Flow cytometry**		
**Human**		
CD31-PE (AC128)	1:40	Miltenyi
CD105-FITC ( SN6)	1:40	Serotec
CD146-PE ( F4-35H7)	1:20	Biocytex
UEA-1-FITC	1:50	Sigma
**Mouse**		
CD31-PE (MEC13.3)	1:20	BD Biosciences
CD34-FITC (RAM34)	1:20	BD Biosciences
CD146-FITC (P1H12)	1:20	Chemicon
Tie-2-PE (1E11DH)	1:50	Millipore

### Cord formation on Matrigel

200 μL of Matrigel (12.5 mg/mL, BD Bioscience) at 4°C were transferred to pre-chilled 24-wells culture plates. After gentle agitation to ensure complete coating, plates were incubated for 30 minutes at 37°C to allow the solidification of Matrigel. Human and murine BMVECs were then seeded at a concentration of 6 × 10^4^/well in EndoPM [18]. Cord formation was detectable after 5-7 hours of incubation in ten fields randomly photographed.

### Acetylated low-density lipoprotein uptake

To determine the uptake of Dil labeled acetylated low-density lipoprotein, cells were incubated with 10 mg/mL Dil-Ac-LDL (Molecular Probes, Invitrogen) at 37°C for 4 hours. The cells were washed with PBS and mounted with Fluorsave™ (Calbiochem-Millipore, Temecula, CA, USA). The slides were analyzed using a Nikon Eclipse TE300 inverted microscope equipped with a Zeiss Axiovision device camera.

### Vascular permeability assay

2 × 10^4^ BMVECs were plated on collagen coated insert of Transwell (Corning Life Science, Union City, CA, USA) with membrane filter (0.4-μm pore size) in EndoPM medium in the upper and in the lower chamber. 5 days before assay the cells were grown in EndoPM medium without Hormone Mix, until they have reached confluence (day 3 = semiconfluence). The confluence was determined by hematoxylin-eosin staining of sentinel well. FITC-dextran (4 μL, 25 mg/mL initial concentration) (Sigma-Aldrich, St.Louis, MO, USA) was filled to the insert. Every 30 min, 50 μL of medium was collected from the lower chamber. The aliquots were diluted to 1 mL with 1× PBS. 100 μL of each diluted sample were transferred into 96-well black plates and the fluorescent content at 492/520 nm absorption/emission wavelengths for FITC-dextran was measured.

Transfer permeability of the EC monolayer correlates with the fluorescent intensity in the lower chamber. No fluorescent intensity is detected in the lower chamber once the EC monolayer reaches confluency [19].

### Detection of endothelial-specific gene transcripts by qualitative PCR and quantitative real time PCR

After 60 DIV in EndoPM, endothelial genes expression of human and murine BMVECs was analyzed by means of qualitative PCR and quantitative Real Time PCR and compared with HMVECs.

#### Total RNA extraction and cDNA synthesis

Total RNA was extracted from human and mouse BMVECs cultivated in EndoPM or EBM (as positive control), using TRIzol® reagent (Invitrogen, Carlsbad, CA, USA) and subsequently treated with DNase I (Ambion, Austin, TX, USA). RNA from each sample was reverse transcribed with random hexamer primers reported in Table 3 and Superscript III reverse transcriptase (Invitrogen), following the manufacturer’s.

**Table 3:** RT-PCR primer sequences and expected product size

**Primers**	**Sequences (5’-3’)**	**Product size (bp)**	**Annealing T (°C)**
**Human**			
CD31-f	GCT GTT GGT GGA AGG AGT GC	645	49
CD31-r	GAA GTT GGC TGG AGG TGC TC		
vWF-f	CAC TGA CAC CTG AGT GAG AC	696	60
vWF-r	GTT CGT CCT GGA AGG ATC GG		
Tie-2-f	GGC CGC TAC CTA CTA ATGA AG	550	50
Tie-2-r	CGT GAT TGA CAC TGG ACA TAA		
P-gp-f	GCA AAG CTG GAG AGA TCC TCA CCA	305	60
P-gp-r	CAA CAT TTT CAT TTC AAC AAC TCC TGC		
GAPDH-f	CGG AGT CAA CGG ATT TGG TCG TAT	307	58
GAPDH-r	AGC CTT CTC CAT GGT GGT GAA GAC		
**Mouse**			
CD31-f	GTC ATG GCC ATG GTC GAG TA	260	55
CD31-r	CTC CTC GGC GAT CTT GCT GAA		
vWF-f	TGT TCA TCA AAT GGT GGG CAG C	270	62
vWF-r	ACA GAC GCC ATC TCC AGA TTC A		
Tie-2-f	GGA CAG TGC TCC AAC CAA ATG	154	60
Tie-2-r	GAC GGA AAT GTT GAA AGG C		
P-gp-f	TGC TTA TGG ATC CC	435	55
P-gp-r	TTG GTG AGG ATC TCT CCG GCT		
GAPDH-f	GTC GGT GTG AAC GGA TTT G	280	55
GAPDH-r	TAG ACT CCA CGA CAT ACT CAG CA		

#### Real-time quantitative PCR

For the detection of genes related to EC biology, Real-Time PCR was performed using the human and mouse Endothelial Cell Biology RT^2^*Profiler*™ PCR Array according to manufacturer’s instructions (SA Bioscience Corporation, Frederick, MD, USA). Pathway-focused gene expression analysis was performed with the PCR Array System and the PCR Array Data Analysis Web Portal. Each Endothelial Cell Biology RT^2^*Profiler*™ PCR Array was performed on separate cDNAs at least three times.

### *In vivo* angiogenic assay

Mice were obtained from Charles River Italy, Calco (LC), Italy. CD1 mice, 8-10 weeks of age, were anesthetized with a solution of Tribromoethanol (Avertin®), at a dose of 0.5 mg/g body weight. 2 × 10^5^ human BMVECs in 500 μL of HA hydrogel (Extracel™-X Hydrogel Kit, Glycosan BioSystems, Salt Lake City, UT, USA) were injected with 18-gauge needle in the abdominal quadrant keeping mice in the Trendelenburg position (head down and legs up) to decrease the risk of bowel perforation. Control samples were taken from sham operated mice treated only with HA hydrogel (Glycosan). Two weeks after transplantation, the abdominal quadrants were removed and the isolated patches were fixed in 4% paraformaldehyde. Serial sections of 20 μm, cut using a vibratome, were processed by immunofluorescence. To detect human cells, tissue sections were stained with human nuclear antigen (hNAg). Cell engraftment in the abdominal quadrant was evaluated by immunohistological analysis with UEA-1 and Draq5 (Table 2). Ten fields from 20 tissues sections were randomly selected and the images were acquired with Leica TCS SP2 AOBS (Leica Microsystems, Heidelberg, Germany) confocal laser scanning microscope.

### Statistical analysis

Each experiment was performed in triplicate, values are expressed as mean ± SD. Comparisons of parameters were performed using the Student's t-test. Values of P < 0.05 (*), P < 0.01 (**) and P < 0.001 (***) were considered statistically significant.

## Results

### BMVECs growth in EndoPM and EBM

**Figure 1:**
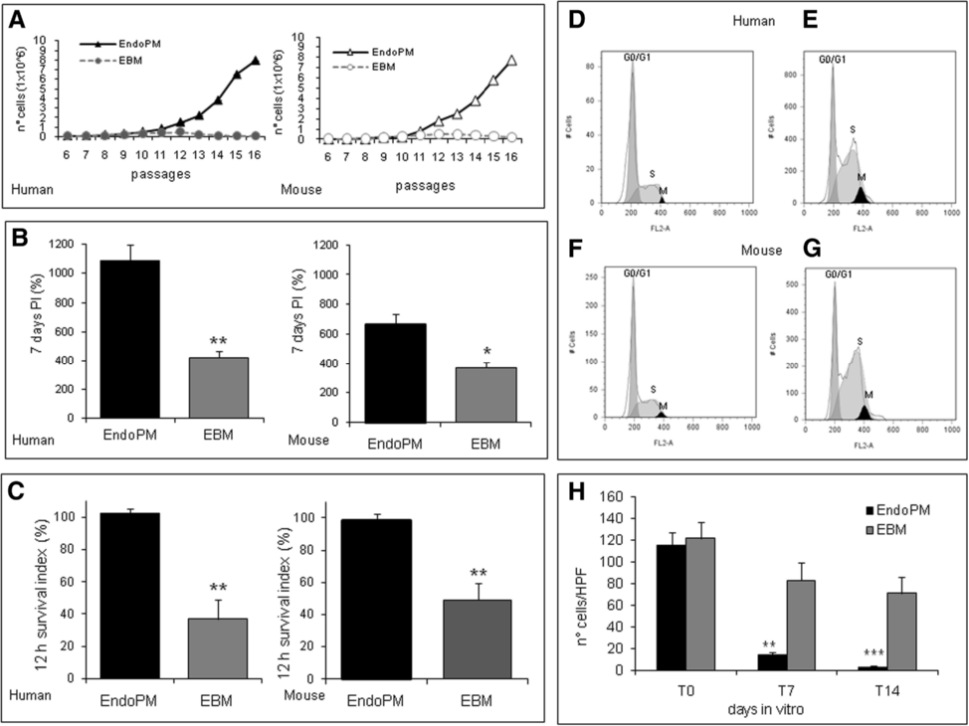
**Culture characteristics of human and murine BMVECs. A**) Growth curve of human and mouse BMVECs cultured in EndoPM (black line) or complete EBM (gray lines); **B**) proliferation index of human and mouse BMVECs cultured in EndoPM (black bars) or EBM (gray bars) at 7 DIV; **C**) survival index of human and mouse BMVECs cultured in EndoPM (black bars) or EBM (gray bars) 12 hours after culture; **D**-**G**) cell cycle profiles of human and mouse BMVEC growth in EBM (**D**, **F**) or EndoPM (**E**, **G**). Histograms are representative of one of four independent experiments. **H**) Co-culture of NHDF and BMVECs in EndoPM (black bars) or EBM (gray bars) after 0, 7 and 14 days of culture. Bars represent the number of CFDA-SE positive cells (NHDF) per field (Higer power field - HPF). * P < 0.05; **P < 0.01; ***P < 0.001.

Human and mouse BMVECs cultured in EndoPM or complete EBM were examined for their proliferation rate by growth curve, proliferation index and survival index (Figure 1). Results indicated that human and mouse cells cultured in EndoPM, compared to EBM culture condition, showed a significant increase of growth rate (Figure 1A), proliferation (Figure 1B) and survival (Figure 1C). The mean ± SD of repeated experiments, performed with different cells lines, are reported in Table 1.

### Cell cycle

Cell cycle was evaluated using a flow cytometer after overnight synchronization in starvation medium. The data showed that EndoPM facilitated the mitosis in ECs by speeding the time of duplication. Following appropriate synchronization time, starved cells were treated with complete EndoPM or EBM medium for 5h or overnight. In all the samples examined, the population of cells in mitosis was greater after restart in EndoPM than EBM.

The results are reported in Figure 1D-G and indicate that after overnight incubation in starvation medium the mean % ± SD of G0/G1 of human cells was 66.4% ± 0.9; S phase was 2.9% ± 1.2; M phase was 17.8% ± 3.3; the mean ±SD of G0/G1 of murine cells was 53.8% ± 0.7; S phase was 2.7% ± 1.6; M phase was 19.1% ± 2.6.

For human cells, after a further overnight restart with complete EBM medium, the mean % ± SD of G0/G1 was 49.7% ± 9.1; S phase was 43.2% ± 6.5; M phase was 2.6% ± 0.1 (Figure 1D); while restarting with complete EndoPM medium induced a mean % ± SD of G0/G1 of 32.9% ± 0.4; S phase of 66.3% ± 9.0; M phase of 4.5% ± 0.7 (Figure 1E). For mouse cells, after further overnight restart with complete EBM medium, the mean % ± SD of G0/G1 was 42.5% ± 10.1; S phase was 38.2% ± 7.0; M phase was 2.2% ± 0.2 (Figure 1F); while restarting with complete EndoPM medium induced a mean ±SD of G0/G1 of 33.2% ± 0.5; S phase of 67.2% ± 7.9; M phase of 4.4% ± 0.5 (Figure 1G).

These data suggest that EndoPM induced a G1 phase decrease compared with complete EBM, and was accompanied by a significant increase of cells in the S phase and a slight increase of M phase cells (P < 0.05 for both cells type). Figure 1 (D-G) is representative of repeated experiments performed with different cells lines.

### Co-culture of BMVECs and NHDF

To test the capability of EndoPM on the inhibition of a contaminant cell population, (e.g. fibroblasts), co-cultures of BMVECs and normal human dermal fibroblasts (NHDF) were produced by pooling both cell types at the ratio BMVECS:NHDF = 3:1. 24 h after seeding the mean densities of human BMVECs and NHDF were approximately equal in co-culture (Figure 1H). After 24 h of co-culture, the number of NHDF cells was stable. NHDF cells decreased after 7 and 14 days of culture. During co-culture condition, a significant reduction of NHDF density under the influence of EndoPM was detected compared with complete EBM culture. The reduction of NHDF cells after culture in EndoPM was approximately 95% after 14 days of culture (Figure 1H).

### Expression of endothelial cell phenotype and morphology of EndoPM cultured cells

**Figure 2:**
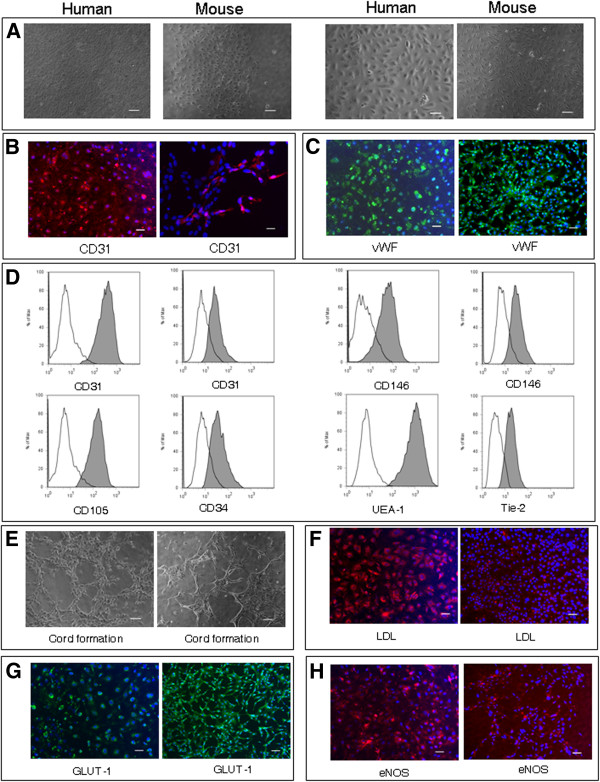
**Characterization and functional features of human and murine BMVECs.** (**A**) Phase contrast micrographs of confluent monolayers of human (left image) and murine (right image) BMVECs. BMVECs present the typical “cobblestone appearance”. Scale bar, 100 μm and 200 μm for human BMVECs and murine BMVECs. **B**) Human (left image) and murine (right image) BMVECs showed a clear cytoplasmic staining for CD31. Scale bar, 50 μm. **C**) Human (left image) and murine (right image) cells displayed an intense positive immunofluorescence for vWf. Scale bar, 50 μm. **D**) Flow cytometric analysis of BMVECs. Human BMVECs resulted positive (gray histograms) for CD31 (left graph), CD105, CD146 (left gaph), UEA-1 staining; murine BMVECs resulted positive for CD31 (right graph), CD34, CD146 (right graph) and Tie-2 staining. White histograms represent the isotype controls of each antibody. **E**) Capillary tube-like structure produced by human (left image) and murine (right image) BMVECs, 7 h after plating onto Matrigel. Scale bar, 100 μm. **F**) LDL-uptake assay on human (left image) and murine (right image) BMVECs. Scale bar, 50 μm. **G**) Human (left image) and murine (right image) BMVECs were labelled for GLUT-1. Scale bar, 50 μm. **H**) Immunofluorescence for eNOS in human (left image) and murine (right image) BMVECs. Scale bar, 50 μm. All nuclei were counterstained with DAPI (blue). One representative of three independent experiments performed in blind is shown for each figure.

BMVECs cultured in EndoPM showed phenotypical and morphological stability during 12 months of culture and no evidence of overgrowth by contaminating cells was found. As seen by phase-contrast microscopy, EndoPM cultured BMVECs formed a monolayer with a cobblestone-like morphology composed of density-inhibited cells, which were not senescent, but still proliferative when split (Figure 2A).

In order to confirm the endothelial phenotype of BMVECs, several EC markers were examined by immunocytochemistry and FACS analysis.

Immunofluorescence analysis revealed strong positivity for CD31 (Figure 2B) and von Willebrand Factor (vWF) (Figure 2C). In addition, FACS analysis by multiple surface epitopes showed high expression of CD31 (human 99.42% ± 0.96; mouse 84.88% ± 16.48), CD146 (human 97.42% ± 4.3; mouse 84.19% ± 11.93), human CD105 (97.96% ± 3), mouse CD34 (82.89% ± 17.64), Ulex europaeus agglutinin-1 (UEA-1) (99.9% ± 0.07) and mouse Tie-2 (84.37% ± 3.48) (Figure 2D).

### Functional assays for BMVECs

To determine whether EndoPM cultured BMVECs were able to show angiogenic function, 6 × 10^4^ cells were plated on Matrigel and the cultures were examined for capillary tube-like structure formation. BMVECs exhibited an angiogenic response within 5-7 h and migrated from the evenly distributed monolayer of cells to form net-like, capillary tube-like structures with open areas with no cells around (Figure 2E). This was observed for every sample of human and murine BMVECs, with no major differences observed.

Using immunofluorescence staining, *in vitro* endothelial functionality was demonstrated on BMVECs by evaluating: uptake of Dil labeled acetylated low-density lipoprotein (Dil-Ac-LDL) (Figure 2F), expression of Glucose transporter 1 (GLUT-1) (Figure 2G) and positivity for endothelial nitric oxide synthase (eNOS) (Figure 2H). A similar uptake of Dil-Ac-LDL (intense punctate staining in the perinuclear region), expression of GLUT-1 and eNOS were detected in human and murine population cultured in complete EBM condition. Overall, these observations further indicate that human and murine BMVECs cultured in EndoPM retain key phenotypic features of ECs, despite serial time-passaging and culture *in vitro*.

**Figure 3:**
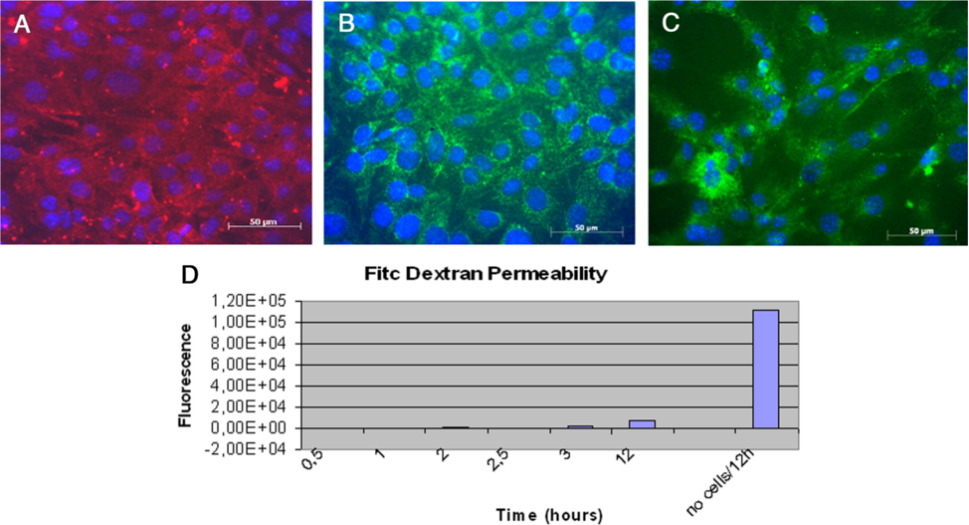
**Tight junctions markers and Vascular Permeability Assay of BMVECs.** Murine BMVECs were isolated and cultured in EndoPM medium, then incubated 5 days in EndoPM medium without Hormone Mix. Immunofluorescent staining of confluent human brain microvascular endothelial cell monolayers for VE-cadherin (**A**), Claudin-5 (**B**), β-catenin (**C**). Nuclei were stained with DAPI. Scale bar = 50 μm. **D**) FITC-dextran permeability of the endothelium monolayer formed after isolation of BMVECs by means of the described protocol. Cells cultured in EndoPM medium and then plated in appropriate transwells strongly reduce macromolecules permeability throughout the barrier over 12 hours, as functional test of vascular permeability. All the experiments were repeated at passages n. 5, 10 and 15 without significant differences in the obtained results.

In addition, cells resulted positive for functional markers of cellular junctions as adherent junctions VE-Cadherin and β-catenin and tight junction Claudin-5, after 5 days culture in EndoPM medium without Hormone mix (Figure 3A, B, C).

### Vascular permeability assay

Macromolecules (FITC-dextran) are used to examine the permeability of the endothelium monolayer formed after isolation of BMVECs by means of the described protocol. Although the readout of this assay is not sufficient to draw conclusions, it is clear that cells cultured in EndoPM medium and then plated in appropriate transwells strongly reduce macromolecules permeability throughout the barrier over 12 hours, as functional test of vascular permeability (Figure 3D).

### BMVEC expression of endothelial genes

**Figure 4:**
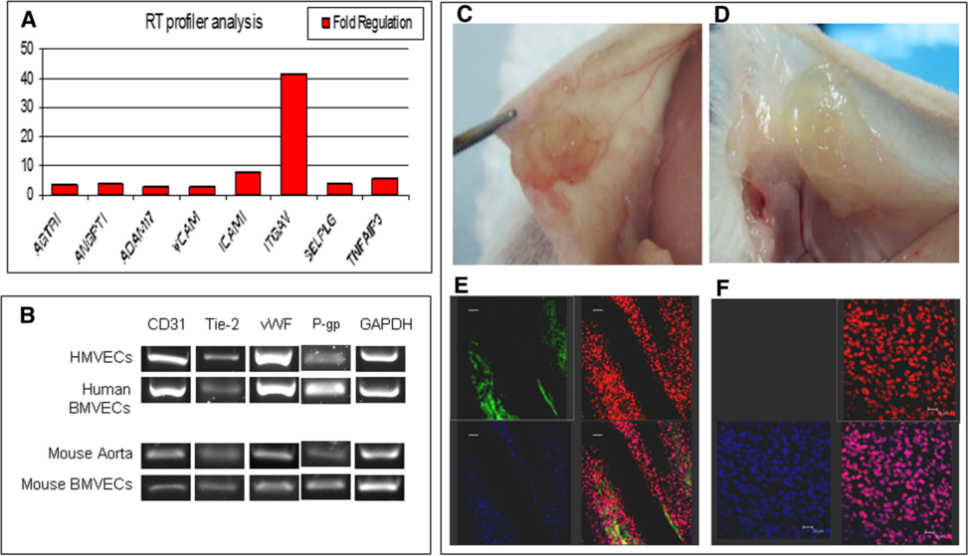
**Gene expression profile and *in vivo *engraftment. A**) RT^2^ profiler of human BMVECs cultured in EndoPM in comparison with cells cultivated in complete EBM; **B**) Endothelial gene expression of human and murine BMVECs analyzed by means of qualitative PCR. C-D) *in vivo* engraftment: photographs of the abdominal quadrant of CD1 mice that received **C**) or did not receive **D**) human BMVECs cultured in EndoPM; **E**) Grafted human BMVECs abdominal quadrant is used for immunostaining. Cells were immunoreactive for UEA-1 (green) 2 weeks after transplantation. The presence of live human ECs was confirmed by Draq5 (red) positivity and anti-human nuclei (hNAg, blue) positivity. The lower-right image represents the merge between UEA-1, Draq5 and anti-human nuclei. Scale bar, 50 μm. **F**) The control abdominal quadrant that received vehicle only resulted UEA-1 negative (upper-left image) and Draq5 (red) positive. DAPI was used for nuclear staining. The lower-right image represents the merge between UEA-1, Draq5 and DAPI. Scale bar, 20 μm.

Using RT-PCR assay, molecular analysis of EC biology was performed on BMVECs to compare gene expression between our isolated EndoPM human and mouse BMVECs and EC line cultured in complete EBM. The identified genes were classified into pro-angiogenic and adhesion molecules genes. BMVECs and HMVECs showed similar expression levels of the most representative angiogenic genes: vWF, KDR, FLT-1, VEGF-A, PECAM-1. In addition, an overexpression of pro-angiogenic genes such as angiopoietin (ANGPT1 +4.14 fold increase), AGTR1 (+3.28 fold increase), SELPLG (+4.02 fold increase) and ADAM17 (+2.68 fold increase) was found. Adhesion molecules genes up-regulated included ITGAV (+41.64 fold increase), ICAM1 (+8.05 fold increase), VCAM1 (+2.60 fold increase) and TNFAIP3 (+5.77 fold increase) (Figure 4A). Furthermore, CD31, vWF, Tie-2 and P-glycoprotein (P-gp) transcripts, analyzed by qualitative PCR, were detected in all cultures tested (Figure 4B).

### *In vivo* angiogenic assay

In order to evaluate whether EndoPM cultured human BMVECs could integrate into mouse tissue and if they had the capability to construct new blood vessels, we opted for a xenograft model in the CD1 mouse strain. 2 × 10^5^ cells were engrafted in the abdominal quadrant of CD1 mouse. We analyzed 15 mice divided into two groups: group 1 (n = 10) underwent transplantation with EndoPM cultured human BMVECs embedded in hydrogel, group 2 (n = 5) underwent injection of hydrogel without BMVECs. Two weeks after transplantation, macroscopic differences were noticed in mice that received BMVECs in hydrogel (Figure 4C) compared with the mice that received only hydrogel (Figure 4D). Histological examination with human specific antibodies showed that implanted cells were immunoreactive for UEA-1 and for double staining with Draq5 and anti-human nuclei (hNAg), thus confirming the presence of human cells expressing the endothelial marker in many sections (Figure 4E). The specificity of the staining was established by the absence of human UEA-1 and anti human nuclei positive cells in the patch of mice that did not receive endothelial cells (Figure 4F).

## Discussion

Several recent studies have highlighted the importance of research on the brain ECs that form the blood brain barrier (BBB) for the study of the physiology and pharmacology of cerebrovascular diseases [20-22].

In the present study, we describe a newly formulated medium for the culture of brain-derived human and mouse ECs (BMVECs), based on our studies in the isolation, culture and characterization of ECs from different human organs [23,24]. Such invention is suitable for the generation of an *in vitro* model in the field of cerebrovascular diseases’ investigation. It guaranties the isolation and maintenance of BMVECs with high purity, in any laboratory and with minimal equipment and accessories.

In contrast with other methods described in the literature [11,18,21,25], our technique is simply based on the formulation of a low-serum medium called “Endothelial Proliferation Medium” (EndoPM, patent number MI2011A 000201), with the addition of a high concentration of growth factors, but without immunomagnetic selections or gene manipulations. In order to show the efficacy of EndoPM, we considered whether BMVECs isolated and maintained in the newly formulated medium demonstrated both phenotypical and functional endothelial cell markers. EndoPM medium had a significant effect on inducing the selection and proliferation of BMVECs when compared with commercially available EBM medium supplemented with growth factors kit. The cell population cultured in EndoPM formed typical cobblestone patterns consistent with their EC lineage [6]. BMVECs, cultured for more than 60 DIV in EndoPM, continuously maintained endothelial features during the course of this study. Moreover, BMVECs could be cryopreserved, maintaining high viability and showing expression of characteristic markers after thawing (data not shown).

One of the most important features of this technique is the efficiency of purification from fibroblast contamination in our BMVECs culture, as demonstrated in our laboratory by co-culture assays.

This significant effect of EndoPM on BMVECs was confirmed by cell cycle analysis. Data show that cells cultured in EndoPM were able to overcome the G1 restriction checkpoint level more easily than when cultured in complete EBM medium. The G1 phase checkpoint (the restriction point) is controlled mainly by the action of the CKI-p16 (CDK inhibitor p16), a protein that inhibits CDK4/6, ensuring no more interaction with cyclin D1. Its levels are maintained through G1 phase and are required for the initiation of S phase, at which time cyclin D1 levels are automatically reduced to low levels. In this way, cyclin D1 is proposed to serve as an active switch in the regulation of continued cell cycle progression [17]. Analysis on the expression level of cyclins in EBM and EndoPM endothelial cultures are still ongoing.

Human and murine BMVECs cultured in EndoPM expressed all the markers typically used to confirm endothelial lineage [21], such as CD31 and vWF, and were accompanied by rapid formation of capillary-like networks [26], intense endocytosis of Dil-Ac-LDL, eNOS [27], specific transporters (i.e., GLUT-1 [28]) and P-gp [29,30] expression. Moreover BMVECs were able to express markers of the junctional architecture such as Ve-cadherin, β-catenin and Claudin-5, demonstrating their *in vitro* capability to show typical features of BBB. In addition, BMVECs, in confluence culture condition, inhibit macromolecules transcytosis as demonstrated by vascular permeability assay [31].

In order to identify transcriptional differences between cells cultured under these two expansion-media conditions, a combined panel for endothelial phenotype related gene expression profile [32] analysis was carried out by using Real Time PCR. Our data revealed higher expression of genes related to the angiogenetic pathway on EndoPM cultured BMVECs, such as ANGPT1, AGTR1, SELPLG and ADAM17. Gene expression analysis and endothelial markers expression, also after engraftment in CD1 mouse strain, provided further evidence of endothelial origin of our BMVECs isolated and expanded with EndoPM.

We also observed additional beneficial features of EndoPM particularly higher and longer-term proliferation rates of BMVECs without concerns regarding scale and variation. Moreover, phenotypical/functional characteristics of cells were not altered by or after freezing/thawing storage techniques.

## Conclusion

Our results suggest that BMVECs cultivated with our new technique retain numerous endothelial characteristics, and we believe that these cells will provide a promising tool for use in the development of *in vitro* models that simulate cerebrovascular diseases as well BBB pathologies.

Our invention permits studies on human and murine BMVECs with extended lifetime, good viability and purity without alterations to their endothelial features.

Cell lines cultured with our method could be a useful model for studying the biology of cerebral endothelium in the context of neuro-inflammatory, neurodegenerative or infectious diseases and for large-scale screening of CNS drug candidates.

It should be noted that we have just begun application of BMVECs isolated with our method for the studies in cerebrovascular diseases area, and it is expected that further efforts to strengthen our knowledge of their functional properties will add significant value to the cells, which are known to be essential to the establishment of *in vitro* cerebrovascular diseases and BBB models.

Here we demonstrate improved efficiencies for BMVECs isolated specifically from human and mouse brain biopsies. In the future these conditions should be investigated for the isolation of ECs from other body districts, such as adipose, skin or bone marrow cell populations.

## Abbreviations

ECs: Endothelial cells; BMVECs: Brain Microvascular Endothelial Cells; HMVECs: Human Microvascular Endothelial Cells; NHDF: Normal Human Dermal Fibroblast; BBB: Blood Brain Barrier; EndoPM: Endothelial Proliferation Medium; EBM: Endothelial Basal Medium; EGF: Epidermal Growth Factor; FGF-2: Basic Fibroblast Growth Factor; BBE: Bovine Brain Extract; eNOS: endothelial Nitric Oxide Synthase; vWF: von Willebrand Factor; Glut-1: Glucose transport 1; hNAg: human nuclear antigen

## Competing interests

The authors declare that they have competing interests, for patenting the medium EndoPM (number of patent MI2011000201).

## Authors’ contributions

SEN: conception and design of *in vitro* and *in vivo* experiments, collection and/or assembly of data, manuscript writing. GM: participation in murine cells experiments, assembly of data and helped to draft the manuscript. SN: carry out cell cycle analysis, dextran permeability. GI: carry out *in vitro* immunofluorescence assays. SC: participation of collection and assembly of molecular data *in vivo and in vitro*. SB: Collection of samples. SS: transplantation and isolation of *in vivo* patches. EC: carry out flow cytometry analysis. AB: participation of *in vitro* experiments. GA: participation in the design of the study and helped to draft the manuscript. MS: participation in performing *in vitro* experiments. EAP: administrative support, supervisor of paper, final approval of manuscript. All authors read and approved the final manuscript.
